# The Pretty Nurse

**Published:** 1900-03

**Authors:** 


### The Pretty Nurse.

"Professional nurses have no business being so confoundedly good-looking," said a young man who has recently spent several weeks in a local infirmary. "The nurse who was delegated to attend to me while I was laid up was a distractingly handsome girl, with a pure Greek profile, reddish-brown hair—the kind that seems full of little golden tendrils in the sunlight—and eyes as liquid as a fawn's. The first time she put her finger on my wrist my pulse ran up to at least 175, and she took it for granted I had a high fever and dosed me accordingly. I tried repeatedly to lure her into conversation, but she wouldn't be lured. She was strictly business. When I started to pay her compliments she would ask me to put out my tongue, which was an insurmountable obstacle in conversation. I used to lie there with my tongue hanging out, trying to put my whole soul into my eyes, but it was no go. No man can look romantic with half a foot of furry red tongue protruding from his countenance.

"Another way she had of gagging me was by putting the thermometer in my mouth. The last week I was there I proposed to her five times, or, rather, I tried to, but she invariably choked off my declarations my thrusting a thermometer into my mouth. I got so excited one time that I came near swallowing a thermometer worth several dollars. She was a most excellent young woman and had lots of sound common sense, as was evidenced by the fact that she gave me no encouragement whatever."—*New Orleans Times-Democrat.*

**Malignant Syphilis.**—Inject from twelve to fifteen ounces of Hayem's solution by four or five subcutaneous punctures at intervals of five or six days.

| | | |
|---|---|---|
| ℞ | Sodii chloridi.................... | 7 parts. |
| | Sodii phosphat................... | 2 " |
| | Aquæ...............................1,000 | " |

—*Augagneur.*

---

**Alopecia.**—*The Monthly Cyclopedia of Medicine* abstracts the following from an article by Balzer in *La Sem. Med.:* In alopecia one of the following formulæ, after the suggestion made by Richema and M. Stoganovitch, has been found useful:

| | | | |
|---|---|---|---|
| 1. | ℞ | Lactic acid...................... | 4 fluidrachms. |
| | | Distilled water................. | 1 fluidounce. |
| 2. | ℞ | Lactic acid...................... | 2½ fluidrachms. |
| | | Alcohol (60 per cent)......... | 1 fluidounce. |

The application is made with absorbent cotton, by friction, until considerable redness is caused. After successive applications, if crusts form, borated vaselin is applied until normal condition is obtained, and then the lactic acid is resumed. This treatment should be continued until the desired growth of hair is obtained.—*Med. Fortnightlg.*

---

**The Treatment of Hypertrophied Tonsils.**—*Reforma medica* for September 4th recommends:

| | | |
|---|---|---|
| ℞ | Resorcin ....................... | 75 grains; |
| | Water ................ ........ | 15,000 " |

M.

or—

| | | |
|---|---|---|
| ℞ | *B*-naphthol .................... | 7½ grains; |
| | Water .......................... | 15,000 " |

M.

Abundant hot irrigations therewith to be employed morning and evening.—*Exchange.*

**Syrup of Arsenate of Iron.**—The *Riforma Medica* for May 12 credits the following to Griggi:

| | | | |
|---|---|---|---|
| ℞ | Arsenate of sodium............. | $4\frac{1}{10}$ | gr. |
| | Pure ferrus sulphate........... | 5½ | gr. |
| | Citric acid..... .................. | 12 | gr. |
| | Distilled water.................. | 150 | gr. |
| | Syrup............................. | 14850 | gr. |

M.: From two to six teaspoonfuls daily, before meals.—*N. Y. Medical Journal.*

